# Dichlorido(2,3-di-2-pyridyl­pyrazine-κ^2^ *N* ^1^,*N* ^2^)palladium(II)

**DOI:** 10.1107/S1600536811044369

**Published:** 2011-10-29

**Authors:** Kwang Ha

**Affiliations:** a School of Applied Chemical Engineering, The Research Institute of Catalysis, Chonnam National University, Gwangju 500-757, Republic of Korea

## Abstract

The Pd^II^ ion in the title complex, [PdCl_2_(C_14_H_10_N_4_)], is four-coordinated in a distorted square-planar environment defined by two N atoms of a chelating 2,3-di-2-pyridyl­pyrazine (dpp) ligand and two chloride anions. The pyridine ring coordinated to the Pd atom is inclined slightly to its carrier pyrazine ring [dihedral angle = 14.4 (3)°], whereas the uncoordinated pyridine ring is inclined considerably to the pyrazine ring [dihedral angle = 52.2 (2)°]. The dihedral angle between the two pyridine rings is 58.8 (2)°. In the crystal, complex mol­ecules are connected by inter­molecular C—H⋯Cl and C—H⋯N hydrogen bonds, forming a three-dimensional network. Intra­molecular C—H⋯Cl hydrogen bonds are also present.

## Related literature

For related crystal structures of [Pt*X*_2_(dpp)] (*X* = Br, Cl), see: Ha (2011*a* ▶,*b* ▶).
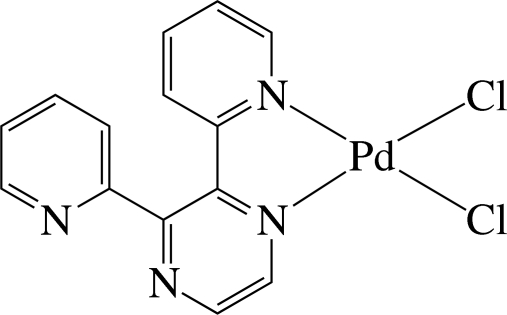


## Experimental

### 

#### Crystal data

[PdCl_2_(C_14_H_10_N_4_)]
*M* *_r_* = 411.56
Triclinic, 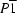

*a* = 8.1681 (10) Å
*b* = 9.5480 (11) Å
*c* = 10.1137 (12) Å
α = 84.543 (2)°
β = 71.400 (2)°
γ = 71.475 (2)°
*V* = 708.81 (15) Å^3^
*Z* = 2
Mo *K*α radiation
μ = 1.68 mm^−1^
*T* = 200 K
0.26 × 0.16 × 0.12 mm

#### Data collection

Bruker SMART 1000 CCD diffractometer
Absorption correction: multi-scan (*SADABS*; Bruker, 2000 ▶) *T*_min_ = 0.748, *T*_max_ = 1.000
5225 measured reflections
3406 independent reflections
2608 reflections with *I* > 2σ(*I*)
*R*_int_ = 0.025

#### Refinement

*R*[*F* ^2^ > 2σ(*F* ^2^)] = 0.046
*wR*(*F* ^2^) = 0.115
*S* = 1.24
3406 reflections
190 parameters
H-atom parameters constrained
Δρ_max_ = 1.18 e Å^−3^
Δρ_min_ = −2.09 e Å^−3^

### 

Data collection: *SMART* (Bruker, 2000 ▶); cell refinement: *SAINT* (Bruker, 2000 ▶); data reduction: *SAINT*; program(s) used to solve structure: *SHELXS97* (Sheldrick, 2008 ▶); program(s) used to refine structure: *SHELXL97* (Sheldrick, 2008 ▶); molecular graphics: *ORTEP-3* (Farrugia, 1997 ▶) and *PLATON* (Spek, 2009 ▶); software used to prepare material for publication: *SHELXL97*.

## Supplementary Material

Crystal structure: contains datablock(s) global, I. DOI: 10.1107/S1600536811044369/tk5006sup1.cif

Structure factors: contains datablock(s) I. DOI: 10.1107/S1600536811044369/tk5006Isup2.hkl

Additional supplementary materials:  crystallographic information; 3D view; checkCIF report

## Figures and Tables

**Table:** 

Pd1—N3	2.035 (5)
Pd1—N1	2.038 (5)
Pd1—Cl2	2.2787 (17)
Pd1—Cl1	2.2860 (17)

**Table:** 

N3—Pd1—N1	80.24 (19)
Cl2—Pd1—Cl1	89.14 (6)

**Table 2:** Hydrogen-bond geometry (Å, °)

*D*—H⋯*A*	*D*—H	H⋯*A*	*D*⋯*A*	*D*—H⋯*A*
C3—H3⋯Cl2^i^	0.95	2.73	3.632 (7)	159
C4—H4⋯Cl1	0.95	2.60	3.216 (7)	123
C8—H8⋯N4^ii^	0.95	2.58	3.529 (9)	178
C9—H9⋯Cl2	0.95	2.62	3.241 (7)	123
C13—H13⋯Cl2^iii^	0.95	2.76	3.530 (7)	139

Symmetry codes: (i) 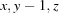; (ii) 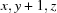; (iii) 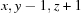.

**Fig. 1.**
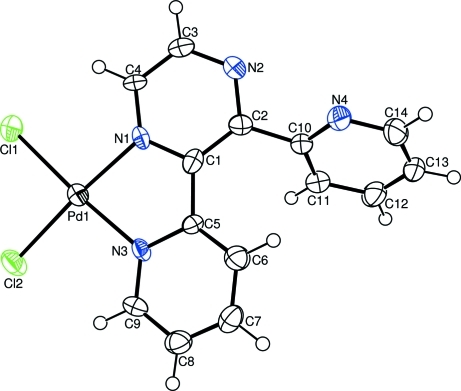
The structure of the title complex, with displacement ellipsoids drawn at the 50% probability level; H atoms are shown as small circles of arbitrary radius.

**Fig. 2.**
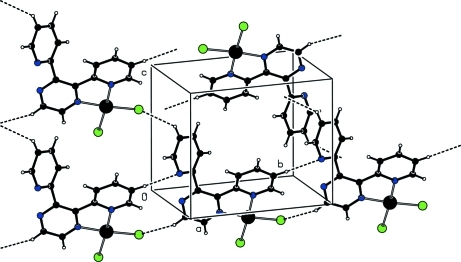
View of the unit-cell contents of the title complex. Intermolecular hydrogen-bond interactions are drawn with dashed lines.

## supplementary crystallographic information

### Comment

In the title complex, [PdCl_2_(dpp)] (dpp = 2,3-di-2-pyridylpyrazine, C_14_H_10_N_4_), the central Pd^II^ ion has a distorted square-planar coordination defined by two N atoms, one from the pyrazine ring and the other from pyridyl ring of the chelating dpp ligand and two Cl^-^ anions (Fig. 1). The complex crystallized in the triclinic space group *P*1, whereas the previously reported analogous Pt^II^ complexes [Pt*X*_2_(dpp)] (*X* = Br, Cl) crystallized in the monoclinic space group *P*2_1_/*n* (Ha, 2011*a*,*b*).

The tight N1—Pd1—N3 chelate angle of 80.24 (19)° contributes the distortion of the square, which results in slightly bent *trans* axes [<Cl1—Pd1—N3 = 174.80 (14)° and <Cl2—Pd1—N1 = 175.50 (15)°]. The pairs of Pd—N and Pd—Cl bond lengths are nearly equal, respectively (Table 1). The pyridyl ring coordinated to the Pd atom is inclined slightly to its carrier pyrazine ring, making dihedral angle of 14.4 (3)°. By contrast, the uncoordinated pyridyl ring is inclined considerably to the pyrazine ring forming a dihedral angle of 52.2 (2)°. The dihedral angle between the two pyridyl rings is 58.8 (2)°. The complexes are connected by intermolecular C—H···Cl and C—H···N hydrogen bonds, forming a three-dimensional network (Fig. 2 and Table 2). There are also intramolecular C—H···Cl hydrogen bonds (Table 2). The molecules stack in columns along the *a* axis and display numerous inter- and intramolecular π-π interactions between the six-membered rings, with a shortest ring centroid-centroid distance of 3.848 (4) Å.

### Experimental

The single crystals of the title complex were obtained as a by-product from the reaction of Na_2_PdCl_4_ (0.2960 g, 1.006 mmol) with 2,3-di-2-pyridylpyrazine (0.2361 g, 1.008 mmol) in MeOH (30 ml). After stirring of the reaction mixture for 20 h at room temperature, the formed precipitate was separated by filtration, washed with MeOH, and dried at 50 °C, to give a yellow powder (0.3560 g). Orange crystals suitable for X-ray analysis were obtained by slow evaporation from an acetone/CH_3_NO_2_ solution of the yellow product.

### Refinement

H atoms were positioned geometrically and allowed to ride on their respective parent atoms [C—H = 0.95 Å and *U*_iso_(H) = 1.2*U*_eq_(C)]. The highest peak (1.18 e Å^-3^) and the deepest hole (-2.09 e Å^-3^) in the final difference Fourier map are located 0.74 Å and 0.88 Å from the N1 and Pd1 atoms, respectively.

### Crystal data

**Table:**

[PdCl_2_(C_14_H_10_N_4_)]	*Z* = 2
*M**_r_* = 411.56	*F*(000) = 404
Triclinic, *P*1	*D*_x_ = 1.928 Mg m^−^^3^
Hall symbol: -P 1	Mo *K*α radiation, λ = 0.71073 Å
*a* = 8.1681 (10) Å	Cell parameters from 2636 reflections
*b* = 9.5480 (11) Å	θ = 2.8–28.2°
*c* = 10.1137 (12) Å	µ = 1.68 mm^−^^1^
α = 84.543 (2)°	*T* = 200 K
β = 71.400 (2)°	Block, orange
γ = 71.475 (2)°	0.26 × 0.16 × 0.12 mm
*V* = 708.81 (15) Å^3^	

### Data collection

**Table:**

Bruker SMART 1000 CCD diffractometer	3406 independent reflections
Radiation source: fine-focus sealed tube	2608 reflections with *I* > 2σ(*I*)
graphite	*R*_int_ = 0.025
φ and ω scans	θ_max_ = 28.3°, θ_min_ = 2.1°
Absorption correction: multi-scan (*SADABS*; Bruker, 2000)	*h* = −10→10
*T*_min_ = 0.748, *T*_max_ = 1.000	*k* = −12→11
5225 measured reflections	*l* = −12→13

### Refinement

**Table:**

Refinement on *F*^2^	Primary atom site location: structure-invariant direct methods
Least-squares matrix: full	Secondary atom site location: difference Fourier map
*R*[*F*^2^ > 2σ(*F*^2^)] = 0.046	Hydrogen site location: inferred from neighbouring sites
*wR*(*F*^2^) = 0.115	H-atom parameters constrained
*S* = 1.24	*w* = 1/[σ^2^(*F*_o_^2^) + (0.*P*)^2^ + 3.5422*P*] where *P* = (*F*_o_^2^ + 2*F*_c_^2^)/3
3406 reflections	(Δ/σ)_max_ < 0.001
190 parameters	Δρ_max_ = 1.18 e Å^−^^3^
0 restraints	Δρ_min_ = −2.09 e Å^−^^3^

### Special details

**Table:**

Geometry. All e.s.d.'s (except the e.s.d. in the dihedral angle between two l.s. planes) are estimated using the full covariance matrix. The cell e.s.d.'s are taken into account individually in the estimation of e.s.d.'s in distances, angles and torsion angles; correlations between e.s.d.'s in cell parameters are only used when they are defined by crystal symmetry. An approximate (isotropic) treatment of cell e.s.d.'s is used for estimating e.s.d.'s involving l.s. planes.
Refinement. Refinement of *F*^2^ against ALL reflections. The weighted *R*-factor *wR* and goodness of fit *S* are based on *F*^2^, conventional *R*-factors *R* are based on *F*, with *F* set to zero for negative *F*^2^. The threshold expression of *F*^2^ > σ(*F*^2^) is used only for calculating *R*-factors(gt) *etc*. and is not relevant to the choice of reflections for refinement. *R*-factors based on *F*^2^ are statistically about twice as large as those based on *F*, and *R*- factors based on ALL data will be even larger.

### Fractional atomic coordinates and isotropic or equivalent isotropic displacement parameters (Å^2^)

**Table:**

	*x*	*y*	*z*	*U*_iso_*/*U*_eq_	
Pd1	0.71773 (7)	0.48277 (5)	−0.10860 (5)	0.02952 (14)	
Cl1	0.6858 (3)	0.43540 (19)	−0.31486 (17)	0.0416 (4)	
Cl2	0.6748 (2)	0.72437 (18)	−0.17098 (19)	0.0416 (4)	
N1	0.7538 (6)	0.2716 (5)	−0.0374 (5)	0.0241 (10)	
N2	0.7697 (8)	0.0024 (6)	0.0868 (6)	0.0384 (13)	
N3	0.7611 (6)	0.5057 (5)	0.0743 (5)	0.0249 (10)	
N4	0.8648 (8)	−0.0406 (6)	0.3339 (6)	0.0398 (14)	
C1	0.7696 (8)	0.2515 (7)	0.0931 (6)	0.0305 (14)	
C2	0.7656 (9)	0.1158 (7)	0.1572 (7)	0.0313 (14)	
C3	0.7645 (11)	0.0272 (8)	−0.0442 (7)	0.0450 (18)	
H3	0.7708	−0.0524	−0.0972	0.054*	
C4	0.7504 (9)	0.1629 (7)	−0.1050 (7)	0.0349 (15)	
H4	0.7381	0.1782	−0.1960	0.042*	
C5	0.7923 (7)	0.3781 (6)	0.1508 (6)	0.0228 (12)	
C6	0.8494 (9)	0.3771 (7)	0.2643 (7)	0.0362 (15)	
H6	0.8776	0.2883	0.3149	0.043*	
C7	0.8667 (9)	0.5053 (8)	0.3064 (7)	0.0368 (15)	
H7	0.9028	0.5056	0.3868	0.044*	
C8	0.8305 (9)	0.6301 (8)	0.2296 (7)	0.0381 (16)	
H8	0.8414	0.7189	0.2558	0.046*	
C9	0.7787 (8)	0.6264 (6)	0.1150 (7)	0.0288 (13)	
H9	0.7541	0.7140	0.0621	0.035*	
C10	0.7479 (8)	0.0837 (7)	0.3079 (6)	0.0290 (13)	
C11	0.6105 (9)	0.1744 (7)	0.4115 (7)	0.0362 (15)	
H11	0.5300	0.2628	0.3891	0.043*	
C12	0.5941 (10)	0.1325 (8)	0.5488 (7)	0.0434 (17)	
H12	0.5014	0.1921	0.6228	0.052*	
C13	0.7126 (11)	0.0043 (8)	0.5770 (7)	0.0461 (19)	
H13	0.7026	−0.0273	0.6704	0.055*	
C14	0.8465 (11)	−0.0774 (8)	0.4673 (8)	0.049 (2)	
H14	0.9305	−0.1649	0.4874	0.059*	

### Atomic displacement parameters (Å^2^)

**Table:**

	*U*^11^	*U*^22^	*U*^33^	*U*^12^	*U*^13^	*U*^23^
Pd1	0.0345 (3)	0.0262 (3)	0.0292 (3)	−0.0109 (2)	−0.0108 (2)	0.00380 (18)
Cl1	0.0574 (11)	0.0411 (9)	0.0321 (9)	−0.0175 (8)	−0.0211 (8)	0.0073 (7)
Cl2	0.0506 (10)	0.0293 (8)	0.0514 (11)	−0.0165 (7)	−0.0235 (9)	0.0126 (7)
N1	0.024 (2)	0.022 (2)	0.024 (3)	−0.008 (2)	−0.006 (2)	0.0076 (19)
N2	0.057 (4)	0.025 (3)	0.036 (3)	−0.013 (3)	−0.019 (3)	0.005 (2)
N3	0.027 (3)	0.022 (2)	0.030 (3)	−0.010 (2)	−0.014 (2)	0.009 (2)
N4	0.055 (4)	0.030 (3)	0.036 (3)	−0.006 (3)	−0.022 (3)	0.001 (2)
C1	0.032 (3)	0.036 (4)	0.022 (3)	−0.008 (3)	−0.009 (3)	0.000 (3)
C2	0.037 (3)	0.024 (3)	0.030 (3)	−0.007 (3)	−0.008 (3)	−0.004 (2)
C3	0.076 (5)	0.032 (4)	0.036 (4)	−0.020 (4)	−0.024 (4)	−0.006 (3)
C4	0.059 (4)	0.024 (3)	0.028 (3)	−0.016 (3)	−0.016 (3)	−0.006 (2)
C5	0.023 (3)	0.023 (3)	0.022 (3)	−0.005 (2)	−0.007 (2)	−0.004 (2)
C6	0.040 (4)	0.032 (3)	0.039 (4)	−0.010 (3)	−0.017 (3)	0.001 (3)
C7	0.034 (3)	0.053 (4)	0.028 (3)	−0.019 (3)	−0.011 (3)	0.000 (3)
C8	0.042 (4)	0.040 (4)	0.032 (4)	−0.018 (3)	−0.004 (3)	−0.007 (3)
C9	0.030 (3)	0.020 (3)	0.037 (4)	−0.009 (2)	−0.008 (3)	−0.002 (2)
C10	0.037 (3)	0.026 (3)	0.029 (3)	−0.013 (3)	−0.013 (3)	0.001 (2)
C11	0.046 (4)	0.032 (4)	0.033 (4)	−0.014 (3)	−0.013 (3)	−0.002 (3)
C12	0.058 (5)	0.050 (4)	0.027 (4)	−0.027 (4)	−0.008 (3)	0.000 (3)
C13	0.081 (6)	0.044 (4)	0.029 (4)	−0.035 (4)	−0.024 (4)	0.009 (3)
C14	0.075 (6)	0.040 (4)	0.055 (5)	−0.025 (4)	−0.046 (5)	0.015 (4)

### Geometric parameters (Å, °)

**Table:**

Pd1—N3	2.035 (5)	C4—H4	0.9500
Pd1—N1	2.038 (5)	C5—C6	1.368 (8)
Pd1—Cl2	2.2787 (17)	C6—C7	1.395 (9)
Pd1—Cl1	2.2860 (17)	C6—H6	0.9500
N1—C4	1.308 (7)	C7—C8	1.363 (9)
N1—C1	1.357 (7)	C7—H7	0.9500
N2—C3	1.336 (8)	C8—C9	1.362 (9)
N2—C2	1.338 (8)	C8—H8	0.9500
N3—C9	1.323 (7)	C9—H9	0.9500
N3—C5	1.377 (7)	C10—C11	1.383 (9)
N4—C10	1.331 (8)	C11—C12	1.385 (9)
N4—C14	1.334 (9)	C11—H11	0.9500
C1—C2	1.398 (8)	C12—C13	1.368 (10)
C1—C5	1.477 (8)	C12—H12	0.9500
C2—C10	1.495 (8)	C13—C14	1.373 (11)
C3—C4	1.367 (9)	C13—H13	0.9500
C3—H3	0.9500	C14—H14	0.9500
			
N3—Pd1—N1	80.24 (19)	N3—C5—C1	114.2 (5)
N3—Pd1—Cl2	95.40 (14)	C5—C6—C7	120.6 (6)
N1—Pd1—Cl2	175.50 (15)	C5—C6—H6	119.7
N3—Pd1—Cl1	174.80 (14)	C7—C6—H6	119.7
N1—Pd1—Cl1	95.27 (14)	C8—C7—C6	118.5 (6)
Cl2—Pd1—Cl1	89.14 (6)	C8—C7—H7	120.8
C4—N1—C1	120.7 (5)	C6—C7—H7	120.8
C4—N1—Pd1	124.3 (4)	C9—C8—C7	119.4 (6)
C1—N1—Pd1	114.8 (4)	C9—C8—H8	120.3
C3—N2—C2	117.3 (6)	C7—C8—H8	120.3
C9—N3—C5	119.8 (5)	N3—C9—C8	122.6 (6)
C9—N3—Pd1	125.0 (4)	N3—C9—H9	118.7
C5—N3—Pd1	114.8 (4)	C8—C9—H9	118.7
C10—N4—C14	117.3 (6)	N4—C10—C11	123.3 (6)
N1—C1—C2	117.9 (6)	N4—C10—C2	115.6 (5)
N1—C1—C5	114.9 (5)	C11—C10—C2	121.0 (6)
C2—C1—C5	127.2 (5)	C10—C11—C12	117.9 (7)
N2—C2—C1	121.4 (6)	C10—C11—H11	121.0
N2—C2—C10	113.5 (5)	C12—C11—H11	121.0
C1—C2—C10	125.1 (5)	C13—C12—C11	119.4 (7)
N2—C3—C4	122.5 (6)	C13—C12—H12	120.3
N2—C3—H3	118.8	C11—C12—H12	120.3
C4—C3—H3	118.8	C12—C13—C14	118.5 (7)
N1—C4—C3	119.8 (6)	C12—C13—H13	120.7
N1—C4—H4	120.1	C14—C13—H13	120.7
C3—C4—H4	120.1	N4—C14—C13	123.5 (7)
C6—C5—N3	118.9 (5)	N4—C14—H14	118.2
C6—C5—C1	126.8 (6)	C13—C14—H14	118.2
			
N3—Pd1—N1—C4	−178.6 (5)	Pd1—N3—C5—C1	−6.1 (6)
Cl1—Pd1—N1—C4	−1.2 (5)	N1—C1—C5—C6	−164.9 (6)
N3—Pd1—N1—C1	6.0 (4)	C2—C1—C5—C6	14.1 (10)
Cl1—Pd1—N1—C1	−176.7 (4)	N1—C1—C5—N3	11.2 (7)
N1—Pd1—N3—C9	173.4 (5)	C2—C1—C5—N3	−169.8 (6)
Cl2—Pd1—N3—C9	−7.8 (5)	N3—C5—C6—C7	3.4 (9)
N1—Pd1—N3—C5	0.3 (4)	C1—C5—C6—C7	179.2 (6)
Cl2—Pd1—N3—C5	179.2 (4)	C5—C6—C7—C8	−1.9 (10)
C4—N1—C1—C2	−5.6 (9)	C6—C7—C8—C9	0.1 (10)
Pd1—N1—C1—C2	170.0 (4)	C5—N3—C9—C8	1.3 (9)
C4—N1—C1—C5	173.5 (5)	Pd1—N3—C9—C8	−171.3 (5)
Pd1—N1—C1—C5	−10.9 (6)	C7—C8—C9—N3	0.1 (10)
C3—N2—C2—C1	−4.7 (10)	C14—N4—C10—C11	−0.1 (10)
C3—N2—C2—C10	172.6 (6)	C14—N4—C10—C2	−176.2 (6)
N1—C1—C2—N2	8.5 (9)	N2—C2—C10—N4	50.6 (8)
C5—C1—C2—N2	−170.5 (6)	C1—C2—C10—N4	−132.3 (7)
N1—C1—C2—C10	−168.5 (6)	N2—C2—C10—C11	−125.6 (7)
C5—C1—C2—C10	12.5 (10)	C1—C2—C10—C11	51.5 (9)
C2—N2—C3—C4	−1.8 (11)	N4—C10—C11—C12	−0.5 (10)
C1—N1—C4—C3	−0.6 (10)	C2—C10—C11—C12	175.4 (6)
Pd1—N1—C4—C3	−175.8 (5)	C10—C11—C12—C13	0.0 (10)
N2—C3—C4—N1	4.6 (12)	C11—C12—C13—C14	1.0 (11)
C9—N3—C5—C6	−3.1 (8)	C10—N4—C14—C13	1.2 (11)
Pd1—N3—C5—C6	170.3 (4)	C12—C13—C14—N4	−1.7 (11)
C9—N3—C5—C1	−179.5 (5)		

### Hydrogen-bond geometry (Å, °)

**Table:**

*D*—H···*A*	*D*—H	H···*A*	*D*···*A*	*D*—H···*A*
C3—H3···Cl2^i^	0.95	2.73	3.632 (7)	159
C4—H4···Cl1	0.95	2.60	3.216 (7)	123
C8—H8···N4^ii^	0.95	2.58	3.529 (9)	178
C9—H9···Cl2	0.95	2.62	3.241 (7)	123
C13—H13···Cl2^iii^	0.95	2.76	3.530 (7)	139

Symmetry codes: (i) *x*, *y*−1, *z*; (ii) *x*, *y*+1, *z*; (iii) *x*, *y*−1, *z*+1.
